# *Salmonella enterica *ssp. *arizonae *infection in a 43-year-old Italian man with hypoglobulinemia: a case report and review of the literature

**DOI:** 10.1186/1752-1947-5-323

**Published:** 2011-07-22

**Authors:** Stefano Di Bella, Alessandro Capone, Eugenio Bordi, Emma Johnson, Maria Musso, Simone Topino, Pasquale Noto, Nicola Petrosillo

**Affiliations:** 1 Second Infectious Diseases Division, National Institute for Infectious Diseases, 'Lazzaro Spallanzani', Via Portuense, 292 00149, Rome, Italy; 2 Microbiology Laboratory, National Institute for Infectious Diseases, 'Lazzaro Spallanzani', Via Portuense, 292 00149, Rome, Italy; 3 Clinical Microbiology Registrar, Sheffield Teaching Hospitals Trust, Sheffield, UK

## Abstract

**Introduction:**

*Salmonella enterica *ssp. *arizonae *is an uncommon human pathogen with serious infections reported in immunocompromised hosts. In Europe, only a few cases have been described. Patients with this infection usually have a history of contact with reptiles or travel abroad. We present a case report of infection in a patient with hypoglobulinemia and a literature review.

**Case presentation:**

We describe the case of a 43-year-old Caucasian Italian man with hypoglobulinemia who presented to our hospital with sepsis and diarrhea. A stool culture yielded *S. enterica *ssp. *arizonae*. Our patient was treated with oral ciprofloxacin and made a full recovery. We also present a review of the cases of *S. enterica *ssp. *arizonae *infections previously reported in Europe.

**Conclusions:**

The majority of infections from *S. enterica *ssp. *arizonae *occur in patients who are immunocompromised. Data from the literature suggests that it may be difficult to eradicate the bacteria and thus, prolonged antibiotic courses are often used. It would be advisable for clinicians to investigate for pre-existing immune dysfunction if *S. enterica *ssp. *arizonae *is isolated. In Italy, although there have only been a few cases, the likely route of transmission remains unclear and requires further surveillance.

## Introduction

*Salmonella enterica *ssp. *arizonae *is an uncommon human pathogen with serious human infections reported in hosts with impaired immune function. *S. enterica *ssp. *arizonae *infections have been well described in patients resident within the southwestern part of the US and in Mexico, whereas in Europe only a few cases have been reported. Patients with this infection usually have a history of contact with reptiles or travel abroad.

We present a case report of *S. enterica *ssp. *arizonae *infection in an adult patient with hypoglobulinemia and literature review of previous cases.

## Case presentation

A 43-year-old Caucasian Italian man was admitted to our hospital presenting with fever, mucoid diarrhea and abdominal cramps for the past 20 days. Associated symptoms included malaise and a 5 kg weight loss during this time. During the previous week he had taken paromomycin along with bacitracin and neomycin, with no clinical improvement.

He had been diagnosed with Hodgkin's disease 15 years previously, which was treated with chemotherapy and autologous bone marrow transplant. Three years ago he had experienced a relapse, which was treated with chemotherapy alone. Since then a residual panhypoglobulinemia had been recorded in our patient. He reported extensive travel to many countries around the world, but not during the last year. He was taking no medications and had no known drug allergies.

On admission he was unwell with fever (38.5°C), hypotension (90/60 mmHg) and signs of dehydration. His lungs were clear and cardiac evaluation was normal. He had abdominal tenderness but there was no organomegaly or masses on palpation. Chest radiograph, abdomen ultrasound and electrocardiogram results showed no abnormalities.

Blood cultures and stool cultures for *Salmonella *spp., *Shigella *spp, *Campylobacter *spp. and *Yersinia *spp. were sent to our laboratory. Fecal examinations revealed presence of fecal occult blood (+++) and many leukocytes. Investigation for parasites and immunofluorescence for *Giardia *were negative. Biochemical analysis showed a normal white blood cell count (4.3 × 10^3 ^cells/mm^3^) but revealed very low levels of immunoglobulins: IgG 91 mg/dL (range 800 to 1500 mg/dL), IgA 4 mg/dL (range 90 to 450 mg/dL) and IgM 1 mg/dL (range 60 to 350 mg/dL). Transaminases, renal function and coagulation studies were normal. Inflammatory markers, including erythrocyte sedimentation rate and C reactive protein, were elevated. Blood culture results were negative and supportive therapy with intravenous fluids was started.

On the sixth day of admission, Gram-negative bacilli were isolated from stool cultures and the bioMérieux VITEK^® ^2 system was used to identify the *S. enterica *ssp. *arizonae*. Phenotyping of this *Salmonella *isolate was also performed by the API 20E system (bioMèrieux), confirming the subspecies.

The bacterium was susceptible to all the tested antibiotics (Table 1). Therefore antimicrobial therapy with oral ciprofloxacin (500 mg every 12 hours) was started, after which there was a rapid improvement in our patient's clinical condition, with complete defervescence and cessation of the diarrhea.

**Table 1:** Tested antibiotics

Antibiotic	Minimal inhibitory concentration, μg/mL	Sensitivity
Amikacin	≤2	S

Amoxicillin/Clavulanate	≤2	S

Ampicillin	≤2	S

Cefepime	≤1	S

Cefotaxime	≤1	S

Ceftazidime	≤1	S

Ciprofloxacin	≤0.25	S

Ertapenem	≤0.5	S

Gentamicin	≤1	S

Imipenem	≤1	S

Levofloxacin	≤0.12	S

Meropenem	≤0.25	S

Piperacillin/Tazobactam	≤4	S

Tobramycin	≤1	S

Trimethoprim/Sulfamethoxazole	≤20	S

S = sensitive; I = intermediate; R = resistant.

Due to the severe humoral immunodeficiency in our patient, ciprofloxacin was continued for a total of 28 days. Our rationale was the evidence in the literature that suggests possible later relapse if short antibiotic treatment courses are used [1]. He continued to improve and fecal cultures taken one week and two weeks after the discontinuation of antibiotic therapy were negative.

## Discussion

*Salmonella *spp. are Gram-negative bacilli and members of the Enterobacteriaceae family. They are documented to be pathogens that cause a spectrum of diseases in humans and animals, including domesticated and wild mammals, reptiles, birds, and insects. *Salmonella *spp. infections are caused by consumption of contaminated food, person-to-person transmission, waterborne transmission and numerous environmental and animal exposures.

*S. enterica *ssp. *arizonae *is one of the less common subspecies of *Salmonella*. Like many non-typhoidal salmonellae, it is mostly found in animal species (commonly reptiles) and only occasionally infects humans. Snakes appear to be important carriers of this bacterium, with as many as 78.8% harboring the organism [2].

*S. enterica *ssp. *arizonae *can be difficult to identify due to their distinguishing biochemical features, which include the ability to utilize malonate, liquefy gelatin and the inability to grow in the presence of KCN (potassium cyanide). Isolation of *S. enterica *ssp. *arizonae *from the stools is difficult as some strains ferment lactose within 48 hours (approximately 15%) and they may be routinely discarded as non-pathogens. However the presence of hydrogen sulfide is an important diagnostic clue during routine screening [3].

This *Salmonella *isolate did not ferment lactose within the first 24 hours so it was further investigated as a *Salmonella *species. However, it is prudent, particularly in patients who are immunosuppressed presenting with fever and diarrhea, that coliforms of potential significance are identified where possible. The use of commercial identification kits or automated systems such as VITEK 2, may be necessary.

Since it is becoming increasingly common to keep reptiles as pets, it appears that the incidence of infection with *S. enterica *ssp. *arizonae *is increasing [1]. The organism is part of the normal reptile intestinal flora but can cause disease in monotremes, turkeys, chickens, goats, and humans [4]. *S. enterica *ssp. *arizonae *enteritis or systemic infections have been well described in patients resident in the southern states of the USA [5], whereas in Europe it is much rarer, with only a few cases reported in the literature [4,6-17]. Many cases reported in the US-Mexican border region were related to the use of rattlesnake products (capsules composed of a powder of dried, crushed snake) as an alternative form of medical treatment [5]. This is a common practice in Mexican folk remedies. Rattlesnake capsules are easily obtained in Mexico without a prescription [5].

Most cases of invasive *S. enterica *ssp. *arizonae *infection have been either in younger patients or those with underlying diseases including collagen vascular diseases, malignancy, organ transplantation and HIV infection [18]. This case highlights, once again, the association between the immunocompromised host and increased susceptibility to *S. enterica *ssp. *arizonae*.

We performed a review of the literature available using the PubMed database, searching for cases of *S. enterica *ssp. *arizonae *infections reported in Europe. We found 16 articles from 1992 to 2010. Features of the reported cases are presented in Table 2[4,6-17].

**Table 2:** European cases of *Salmonella enterica *ssp. *arizonae *infection

Reference (first author/year)	Country	Type of infection (no. of cases)	Possible exposure	Specimens	Underlying conditions
Aiken, 2010 [5]	UK	Unknown (3)	Reptiles (two patients), not reported (one patient)	Not reported	Not reported

Schneider, 2009 [6]	France	Septic arthritis (1)	Snake	Synovial fluid	Young age

Bertrand, 2008 [7]	Belgium	Not reported (3)	Snakes (three patients)	Not reported	Young age (two patients), dialytic treatment (one patient)
	
	Netherlands	Not reported (16)	Reptiles (most patients)	Not reported	Not reported
	
	Germany	Not reported (2)	Snakes (two patients)	Not reported	Young age (one patient), unknown (one patient)
	
	Ireland	Not reported (1)	Snake	Not reported	Young age

Starakis, 2007 [8]	Greece	Endocarditis (1)	Vegetables contaminated with turtles feces	Blood	Sickle cell disease, secondary hemochromatosis

Ozdemir, 2006 [9]	Turkey	Sepsis (1)	Unknown	Blood	AIDS

Foster, 2005 [10]	UK	Gastroenteritis (1)	Reptiles	Stools	Young age

Salavert, 2002 [11]	Spain	Abdominal abscess (1)	Veterinarian patient	Pericardial effusion, pericardial biopsy	Obesity

Catani, 2002 [12]	Italy	Pericarditis (1)	Travels	Pus	Chronic renal failure on dialytic treatment

Carfagna, 1998* [13]; Galiè, 1997* [14]	Italy	Severe sepsis (1)	Travels	Blood	Idiopathic CD4+ lymphocytopenia

Carfagna, 1998 [13]	Italy	Septic shock (1)	Travels	Brain and lungs, autoptic specimens	Chronic lymphatic leukemia on cytostatic treatment

Sanyal, 1997 [15]	UK	Gastroenteritis (1)	Snakes	Stools	Young age, Netherton's syndrome

Buck, 1997 [16]	UK	Gastroenteritis (1)	Reptiles (snake)	Stools	Young age

Hall, 1992 [17]	UK (66 cases from 1966 to 1990)	Enteritis (55), symptomless (6), unknown (3)	Travels 23 patients (35%), terrapins two patients, snakes 11 patients	Blood (two patients), stools (66 patients)	49% of infections occurred in babies and young children

*Same clinical case.

In the cases reported in Europe, *S. enterica *ssp. *arizonae *infections are frequently associated with reptile exposure and underlying diseases, similar to those found in North-Central America. However, in Italy only three cases of this infection have been reported but none described any contact with reptiles.

This is the fourth case reported in Italy and, indeed, our patient also had no history of contact with reptiles. In a study conducted in UK from 1966 to 1990, 12 isolations of *S. enterica *ssp. *arizonae *were derived from human foods and, among these, seven isolates were from imported Italian pasta [17]. A recent study detected *S. enterica *ssp. *arizonae *from Pecorino Abruzzese, a traditional cheese produced in Central Italy [19]. Therefore, it is possible that our patient contracted the bacteria from the ingestion of contaminated food.

*S. enterica *ssp. *arizonae *has been found to be susceptible to commonly prescribed antibiotics in several of the case reports, as was the strain isolated from our patient.

## Conclusions

The isolation of *S. enterica *ssp. *arizonae *is commonly associated with a deficit of the immune status, as in the case of our patient. Indeed, the great majority of infections from *S. enterica *ssp. *arizonae *occur in patients who present with underlying medical conditions.

Data from the literature suggest that, for patients who are immunocompromised, it may be more difficult to eradicate the bacteria and thus prolonged antibiotic courses (> 14 days) are often advisable. The absence of recurrence in our patient suggests that 28 days was an appropriate course length.

In conclusion, although infection from *S. enterica *ssp. *arizonae *is rare, it is most prevalent in patients of a younger age or those with underlying diseases. Therefore, it would be advisable for clinicians to investigate for pre-existing immune dysfunction if *S. enterica *ssp. *arizonae *is isolated. Finally, in Italy, although there have only been a few cases, the likely route of transmission remains unclear and requires further surveillance.

### Consent

Written informed consent was obtained from the patient for publication of this case report and any accompanying images. A copy of the written consent is available for review by the Editor-in-Chief of this journal.

## Competing interests

NP has received industry honoraria for lecturing from Wyeth, GSK, Pfizer, MSD, Novartis, Sanofi Aventis, Janssen Cilag, Carefusion, Astellas, Gilead. All other authors report no conflicts.

## Authors' contributions

SD and MM monitored our patient during hospitalization and analyzed data from the literature. EB isolated and identified the bacterium. AC, PN and ST performed the follow-up of our patient after discharge. EJ was the major contributor in writing the manuscript. NP reviewed the manuscript. All authors have read and approved the final manuscript.
